# The Thorough Removal of Deposits in the Successful Treatment of Pyorrhœa

**Published:** 1895-10

**Authors:** James H. Daly

**Affiliations:** Boston


### THE THOROUGH REMOVAL OF DEPOSITS IN THE SUCCESSFUL TREATMENT OF PYORRHŒA.[1]

BY DR. JAMES H. DALY, BOSTON.

Of the vast number of teeth sacrificed every year, it is estimated that as many are lost from deposits as from caries.

That a large part of these losses might be prevented by proper

---

[1] Read before the American Academy of Dental Science, Boston, January 2, 1895.

treatment on the part of patient or operator, or both, goes without question. Nevertheless, when pyorrhœa exists even in a mild form, to a skilful, practiced eye it is continued and aggravated by deposits; but by far the larger number of operators do not recognize this, and stand idly by and impart the information to their patients that such cases are not curable, and that it is but a question of time when all the teeth will be lost in consequence of the existing state of things. Too true, they will be lost by just such existing facts; but if skilful and care taking cleansing were practised as faithfully as was the filling of caries on the part of the same operators, no such statement would be necessary.

The patient, too, is not altogether free from blame in the matter. In many cases, patients who willingly pay for all other work, look upon the cleansing of their teeth, an operation less pleasant to the operator than almost any other, as of such minor importance that the mass of dentists find it practically impossible to charge anything like a reasonable compensation for their services. Unfortunately, we all have to consider compensation; and no operator, self-respecting, can give that conscientious attention to the case at hand that it requires unless he feels that he is to be compensated by a full appreciation or in current coin of the realm.

The presence of green stain upon the teeth is such an objectionable feature in itself, by its unsightliness, that immediate removal is requested and insisted upon by the patients; but calculi, with its insidious way of attaching itself in out-of-the-way places, infringes upon the tooth-structure in such a gradual way that it is thought to be part and parcel of tooth-substance; and not until in some accidental way a piece becomes dislodged, causing the patient to think a piece of tooth-structure has been broken away, does the mouth receive any attention as regards such deposits, and then, in a great majority of cases, in a very superficial manner.

The thorough cleansing of teeth is not the work of the student, but of the most experienced and careful operator. There is no part of our work that is more important nor where more painstaking and careful manipulation is required. The deposit of nodules of tartar upon the roots of teeth, together with certain conditions of the blood favorable to its development, will cause pyorrhœa; but it is still an open question whether the deposit is the cause or the result of the diseased condition.

Dr. Black says that it is his opinion that any irritation that causes the gum to weep serous fluid will cause this serumal calculus to be deposited.

Professor Miller gives as the cause of pyorrhœa, without any question, three factors,—a constitutional taint, a local irritant, and micro organisms.

Dr. Ingersoll says this calculus—sanguinary is his name for it—is the result of ulceration, and that a suppurative process must precede the formation of the deposit.

Dr. Barrett takes an entirely different view, believing the serumal or sanguinary deposit to be the initial lesion. He believes it to be the result of some special stimulation of the pericemental membrane, and that the deposit is similar in origin to hypercementosis. All, however, agree that, whatever the etiology of pyorrhœa may be, the presence of deposit aggravates and continues it, and that to successfully cope with the disease, every deposit must be removed.

A well-known dentist of Boston, in conversation with me in regard to the etiology of pyorrhœa, said he did not know the cause of it; but he did know that, given the immediate care of a child's teeth from infancy, he would promise that no pyorrhœa would be found in that mouth. The inference was that by absolute cleanliness there would be found no suitable culture-ground for the disease to take root. Wherever patients of mine have been scrupulously clean in the care of their mouth I have had no pyorrhœa to treat, but with the careless it has been a constant fight to keep it in check.

Again I say the removal of deposits is not an easy matter. Suitable instruments are required; they must be of the pushing and also of the pulling kind, and they must be kept sharp, in my opinion, and scrupulously clean. If we but follow the excellent advice and instructions given us in a previous paper read before this Academy by Dr. Potter, there will be no danger of our transmitting disease from one patient to another.

The instruments must also be of such shapes and sizes as will enable the operator to reach every surface of the teeth with little or no harm to the soft tissues, and they must also be small and delicate. By far the larger number of instruments in use by the dentists, as a whole, are much too large, and do more harm by seriously wounding tissue than good in the semi-removal of deposits.

Finally, it seems to me that a thorough instrumentation, combined with knowledge and delicate skill, cannot be urged too emphatically; for if pyorrhœa is to be dealt with in a successful manner, where deposits exist, it is of the utmost importance that they be thoroughly removed.

